# Prevalence and determinants of caesarean section in South and South-East Asian women

**DOI:** 10.1371/journal.pone.0229906

**Published:** 2020-03-12

**Authors:** Vivek Verma, Ramesh K. Vishwakarma, Dilip C. Nath, Hafiz T. A. Khan, Ram Prakash, Omer Abid

**Affiliations:** 1 Departments of Neurology, All India Institute of Medical Sciences, New Delhi, India; 2 Department of Biostatistics, King Abdullah International Medical Research Center, Riyadh, Saudi Arabia; 3 King Saud bin Abdulaziz University for Health Sciences, Riyadh, Saudi Arabia; 4 Ministry of the National Guard—Health Affairs, Riyadh, Saudi Arabia; 5 Assam University, Silchar, Assam, India; 6 The Graduate School, University of West London, London, United Kingdom; 7 Department of Quality Assurance Unit, Eurofins Therapeutics Limited, Bangalore, India; 8 Department of Population Health Research, King Abdullah International Medical Research Center, Riyadh, Saudi Arabia; Anglia Ruskin University, UNITED KINGDOM

## Abstract

**Background:**

Caesarean section is considered a relatively preferable and safe method of delivery as compared to normal delivery. Since the last decade, its prevalence has increased in both developed and developing countries. In the context of developing countries viz., South Asia (the highest populated region) and South-East Asia (the third-highest populated region), where a significant proportion of home deliveries were reported,however, the preference for, caesarean delivery and its associated factors are not well understood.

**Objective:**

To study the caesarean delivery in the South and South-East Asian countries and to determine the factors associated with the preference for caesarean delivery.

**Methodology:**

Demographic and Health Survey Data on from ever-married women of nine developing countries of South and South-East Asia viz., Vietnam, India, Maldives, Timor-Leste, Nepal, Indonesia, Pakistan, Bangladesh, and Cambodia have been considered. Both bivariate and binary logistic regression models were used to estimate the propensity of a woman undergoing for caesarean delivery and to assess the influence of maternal socioeconomic characteristics towards the preference for caesarean delivery.

**Results:**

Obtained results have shown an inclination of caesarean delivery among urban than rural women and are quite conspicuous, but is found to be underestimated mostly among rural women. Caesarean delivery in general is mostly predisposed among women whose baby sizes are either very large or smaller than average, have a higher level of education and place of delivery is private medical institutions. The logistic regression also revealed the influence of maternal socioeconomic characteristics towards the preference for caesarean delivery. Based on nine South and South-East Asian countries an overall C-section prevalence of 13%, but based on institutional births its increase to 19%. The forest plot demonstrated that a significant inclination of C-section among urban than rural regions. In Meta-Analysis, very high and significant heterogeneity among countries is observed, but confirms that in terms of prevalence of C-section all of the countries follow independent pattern.

**Conclusion:**

Study of seven urban and four rural regions of nine South and South- East Asian countries showed, a significant inclination towards the caesarean delivery above the more recent outdated WHO recommended an optimal range of 10–15%and are associated maternal socioeconomic characteristics. In order to control unwanted caesarean delivery, the government needs to develop better healthcare infrastructure and along with more antenatal care related schemes to reduce the risks associated with increased caesarean delivery.

## 1. Introduction

Caesarean section in developed and developing countries[1–3], are considered as the mostpreferred method of childbirth. The preference of caesarean section is found to be comparatively high in the last decade[4], and one of the significant reason is attributed to the reduction in mortality risk of death to mother and child during delivery[5],[6]. There are various factors causing an increase in C-section. In most of the developing countries, demographic changes, social and educational improvement have led to an increase in the number of women delaying their pregnancies until getting on their end of fertile life[7]. This social development pooled with accessibility to birth control and infertility treatment has increased the number of women experiencing their first pregnancy only after 35 years of age[8]. The Caesarean section or C-section is a surgical procedure, where delivery proceeds through the abdominal and uterine incision. This procedure is appropriate in situations where vaginal (or normal) delivery is considered life-threatening for the mother and the baby. Although caesarean delivery is considered a relatively safe delivery method but has complications[9] as compared to vaginal birth or a natural method of birth. One of the major issues with the caesarean deliveries other than post-delivery risks and complication is the cost. It increases due to operation and longer stay in hospitals, and that result in an increased financial burden to the families[10]. The most frequently occurring complications during and after a caesarean to mothers and children have already been discussed [11–14]. In thepast, the World Health Organization[15] had suggested that although caesarean section is a safe method, if caesarean rate exceeds the limit of 10–15%, it may not lead to better outcomes. However, that previous suggestion had come under criticism for multiple reasons. The WHO may have changed it's view as it released a statement in 2015 with the headline. WHO recommends that every effort should be made to provide caesarean sections to women in need rather than to achieve any specific rate. Earlier works[16–18] have suggested that if caesarean rate increases above WHO recommended the range, then as a consequence the risk of manifestation of other public health-related problems for both mothers and children will also increase. Some of work[19] have more recently concluded that the 1985 WHO document[15] looked at studies that were incomplete because they examined data from limited sets of countries and often examined outcomes in wealthier countries. In addition, many studies used data from varying years without accounting for heterogeneity across years.

Furthermore, what is being overlooked is that the WHO document[15] looked at correlation only with mortality. Fetal and maternal morbidities were not taken into account for these rates. It is essential to keep foremost in mind that fetal morbidity should be weighed much higher than maternal morbidity as failure to do C-section when indicated can result in babies with profound brain damage which are catastrophic not only for the babies entire future life but also catastrophic for the parental caregivers and the rest of the family.

The rate of caesarean section is usually defined as the fraction of women who adopted caesarean delivery procedure among total childbirths in a specified time period in a specific geographic area. Under the assumption that in this selected area almost all deliveries took place in medical institutions, as the procedure of caesarean delivery is possible their only, then the existing models and estimates of the caesarean rate discussed in the literatures are appropriate. But in developing countries of South and South-East Asia viz., Vietnam, India, Maldives, Timor-Leste, Nepal, Indonesia, Pakistan, Bangladesh and Cambodia, a considerable proportion of child deliveries are carried out at home,known as non-institutional birth(Table 1) and are completely free of risks and complications associated with caesarean deliveries.

**Table 1:** Selected DHS countries, survey years and place of birth of selected South and South-East Asian countries.

Country	Survey Year	Total Births*	Place of Birth	
			At Home (%)	At Hospital (%)
Vietnam	2002	1317	20.2	79.8
Maldives	2009	3817	4.06	95.94
Timor-Leste	2009–10	9806	80.79	19.21
Nepal	2011	5306	62.34	37.66
Indonesia	2012	18021	45.08	54.92
Pakistan	2012–13	11763	50.24	49.76
Bangladesh	2014	7886	76.48	23.52
Cambodia	2014	7165	17.85	82.15
India	2015–16	259627	24.51	75.49

*Total births occurred in last five year preceding the survey

The countries of South and South-East Asia are regions of great social, economic and political diversity. Despite their diversity, countries in this region are attempting to reduce their regional health challenges ([20] and [21]) and promoting safe and healthy maternal and child health, and also encourage deliveries in the supervision of trained health professionals[22], under proper hygienic conditions.

As the caesarean is a surgical procedure and is only possible at medical institutions. Therefore, the present study have focused on the prevalence of caesarean section among women from different South and South-East Asian countries, who have experienced the institutional deliveries, have been investigated. In this study, an attempt has been made to provide a better understanding of the behavioral pattern of among women of these countries towards caesarean section by comparing their residence, educational status, birth parity and choice of place of delivery. Through this study, the dependency and importance of the socio-economic factors on the caesarean section preference for delivery have also been explored.

### The data

Data for this study was obtained from Demographic and Health Surveys (DHS) database on maternal deliveries occurred in nine developing countries viz., Vietnam, India, Mal- dives, Timor-Leste, Nepal, Indonesia, Pakistan, Bangladesh and Cambodia. DHS is a series of nationally representative household surveys that provide information on population, health and nutritional status of mother and child. The study dataset includes only the latest round of data of each selected country. List of selected South and South-East Asian countries and the corresponding survey years are given in Table 1. In the present study, it is assumed that woman corresponding to each country has equal possibility of being experiencing the caesarean delivery; therefore, the analysis is done without incorporating the weight variable.

## 2. Study parameters

The relationship with incidences of caesarean section with some of the associated maternal socio-economic characteristics viz., maternal age, place of residence, level of maternal education, birth order of the child, and type of medical facility that opted for delivery and size of the baby has been modeled. Age as reported subjectively by mother, and grouped into 6 subgroups: 15–19, 20–24, …,40–44. The 45 to 49 age group was not taken into account due to a lack of sufficient data. The type of place of residence is classified as rural and urban. The educational qualification is classified into three classes, primary, secondary and higher. Birth order of the born child is grouped into first, second, third, fourth and fifth or more. The medical institutions that opted for the delivery are grouped as government or private facilities. The size of the child was classified as below average, average and above average, and large. Our interest lies in finding the prevalence of caesarean section in the countries of South and South-East Asia.

### Statistical analyses

Both bivariate logistic regressions and multivariate logistic regression models have constructed separately to estimate the prevalence of caesarean section to a woman based on her socio-economic characteristics, viz., maternal age, place of residence, level of maternal education, birth order of the child, and type of medical facility that opted for delivery and size of the baby. The results obtained from the regression analyses have been presented in terms of the odds ratios (ORs) with 95% confidence interval (CI). Statistical analyses were performed using the Statistical Analysis System (SAS) package, (university edition) and SAS version 9.4, and all other computation is carried out using R (version-3.0.3). Corresponding to each of the associated maternal socio-economic characteristics, the associations with the prevalence of caesarean sections have been examined using binary logistic regression analyses, to examine the effect of socio-economic factors on the odds of caesarean birth and non-caesarean birth. The event of caesarean section during delivery is considered as a dichotomous variable, where if caesarean then denotes'1' and '0' for otherwise.

The database considered for the present study does not contain any individual identifiable information. Due to the unidentified nature of the dataset and no human subject were directly involved in the present study; therefore, this study was exempt from any ethical or Institutional Review Board clearance.

## 3. Results and discussion

### Prevalence of caesarean section

Table 2 presents the rate of caesarean section based on both institutional and non-institutional births, separated by place of residences viz.,rural and urban regions, of South and South-East Asian Countries. A substantial inter-region variation in caesarean rates corresponding to each country has been observed. Obtained results have shown an inclination of caesarean delivery among urban than rural women and are quite conspicuous. Even with additional non-institutional births (Table 2) to institutional births, the rate of caesarean delivery was found in urban part is highest in women of Maldives (39.07%) followed by India (23.64%), Bangladesh (21.82%), Vietnam (21.72%), Pakistan (17.75%) and Indonesia (17.25%), which have crossed the WHO recommended range of 10–15%[19]. The caesarean rate in the rural women of Maldives (30.70%) has only crossed the WHO recommended range. The caesarean rate in women residing in urban regions is five times of rural women in Nepal; three times of rural women of Vietnam, Timor-Leste, Indonesia and Cambodia, and twice of the rural women of India, Pakistan and Bangladesh.

**Table 2:** Cesarean births occurred based on institutional and non- institutional births in different South and South-East Asian countries during the five years preceding the survey.

Country	Urban		Rural		All	
	Births	%	Births	%	Births	%
Vietnam	267	21.72	1050	7.05	1317	10.02
Maldives	494	39.07	3323	30.70	3817	31.78
Timor-Leste	2204	3.18	7602	1.03	9806	1.51
Nepal	1091	11.64	4215	2.78	5306	4.60
Indonesia	8170	17.25	9851	6.96	18021	11.63
Pakistan	4970	17.75	6793	8.05	11763	12.15
Bangladesh	2488	21.82	5398	10.10	7886	13.80
Cambodia	1950	12.97	5215	4.31	7165	6.67
India	61379	23.64	198248	10.68	259627	13.74

To obtain a better estimate for the prevalence of caesarean deliveries in each of the selected South and South-East Asian Countries, the population is classified into two disjointed sub-populations based on their place of delivery viz., institutional or non-institutional. Here, institutional deliveries referred to those deliveries occurred at any private or government medical institutions, whereas births occurred other than any medical institutions, are considered as non-institutional births. To visualize the prevalence of caesarean section and the population at risk, only institutional deliveries have been taken into account for investigation.

Results based on institutional delivery, Table 3 shows that the rate of caesarean delivery found is highest among the urban women of Bangladesh (62.88%) followed by Maldives (39.18%), Pakistan (27.79%), India (27.28%), Indonesia (22.49%), Vietnam (22.05%), and Nepal (17.47%) that have crossed the WHO recommended range of 10–15%. Among the rural women, the caesarean rate is on the higher side of Bangladesh (54.78%) followed by Maldives (32.21%), Pakistan (20.46%) and Indonesia (18.79%), which have crossed the WHO recommended range. The overall caesarean rate in women of five countries viz., India, Maldives, Indonesia, Pakistan, andBangladesh, have found more than 15%.

**Table 3:** Caesarean births occurred based on institutional births in different South and South-East Asian countries during the five years preceding the survey.

Country	Urban		Rural		All	
	Births	%	Births	%	Births	%
Vietnam	263	22.05	788	9.01	1051	12.27
Maldives	490	39.18	3167	32.21	3657	33.14
Timor-Leste	941	7.44	943	8.27	1884	7.86
Nepal	727	17.47	1271	9.21	1998	12.21
Indonesia	6246	22.49	3634	18.79	9880	21.13
Pakistan	3174	27.79	2674	20.46	5848	24.44
Bangladesh	862	62.88	993	54.78	1855	58.54
Cambodia	1846	13.71	4039	5.57	5885	8.12
India	53200	27.28	142797	14.82	195997	18.20

**Fig 1:**
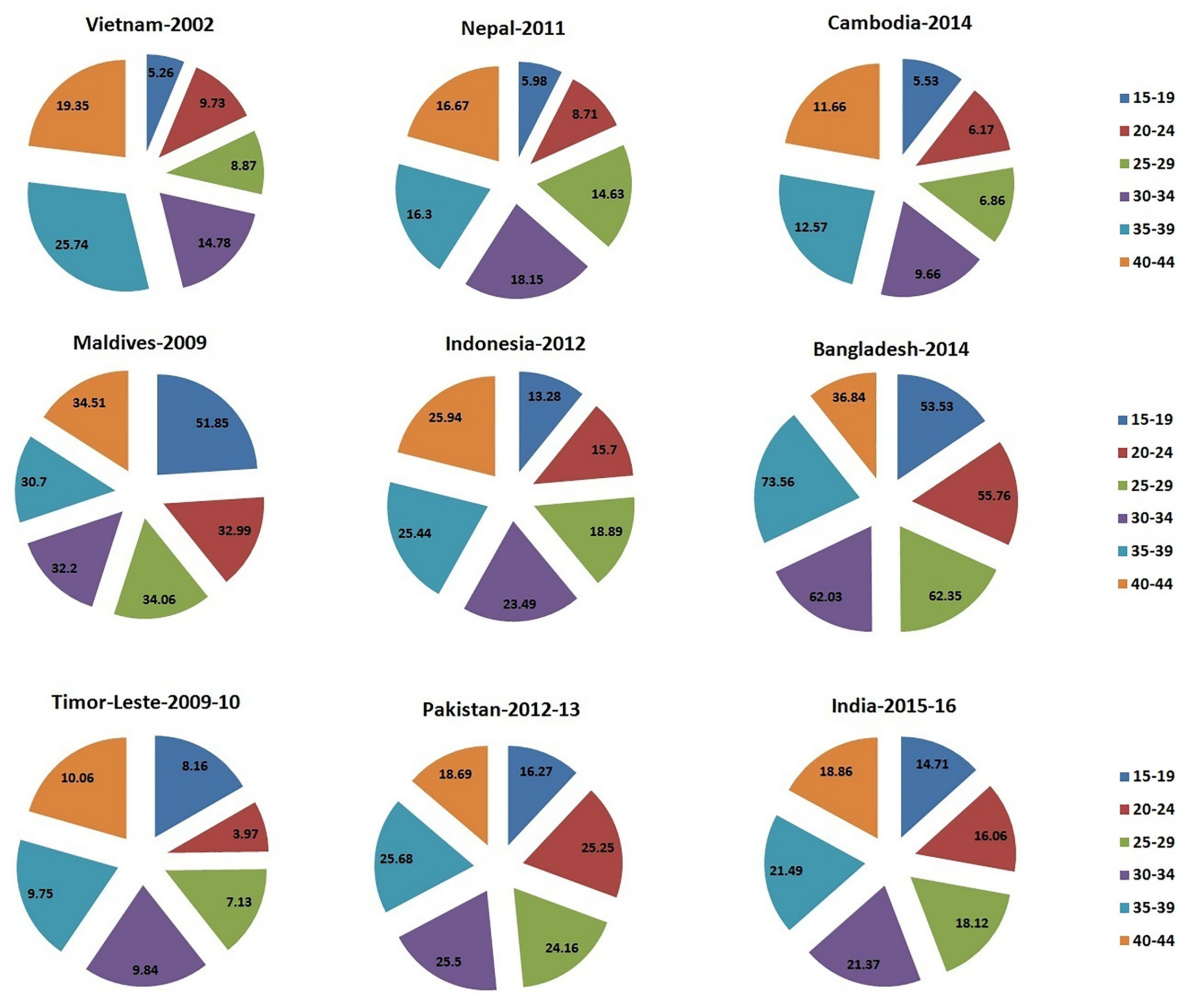
Caesarean rate based on mothers’ age-interval in South and South-East Asian countries.

**Fig 2:**
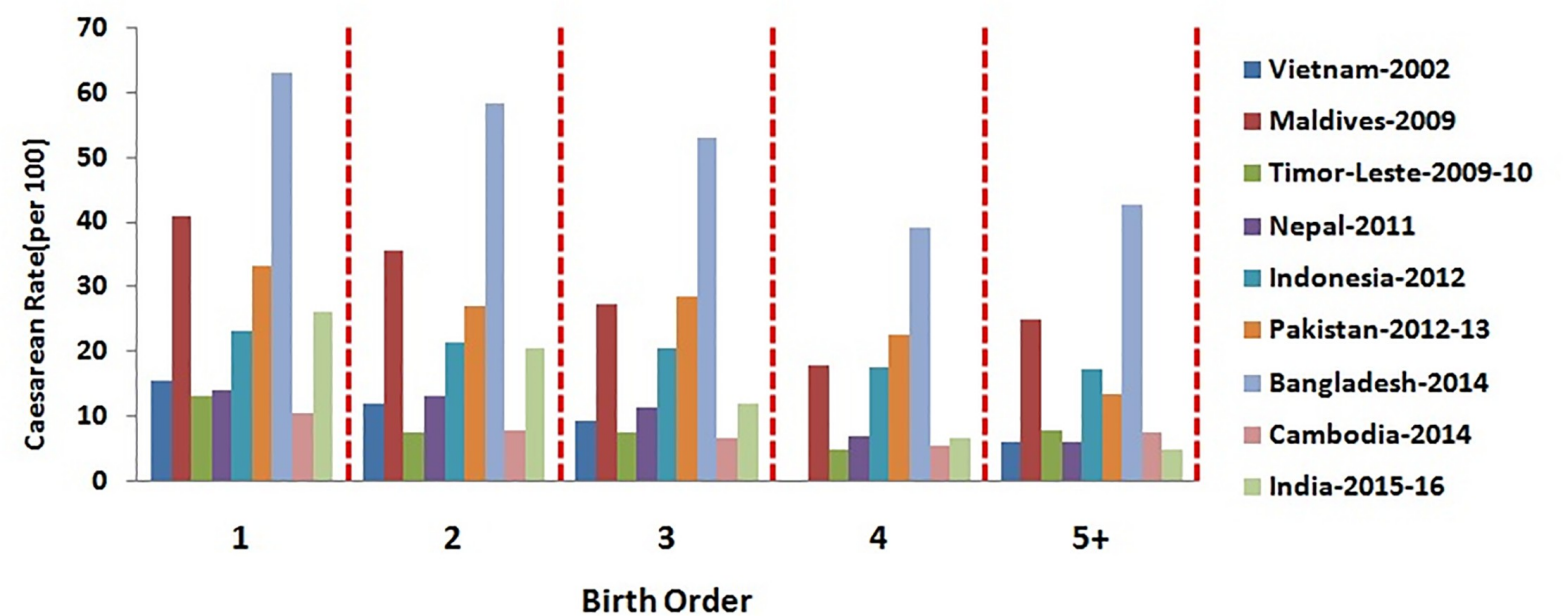
Caesarean rate based on birth order of the child in South and South-East Asian countries.

**Fig 3:**
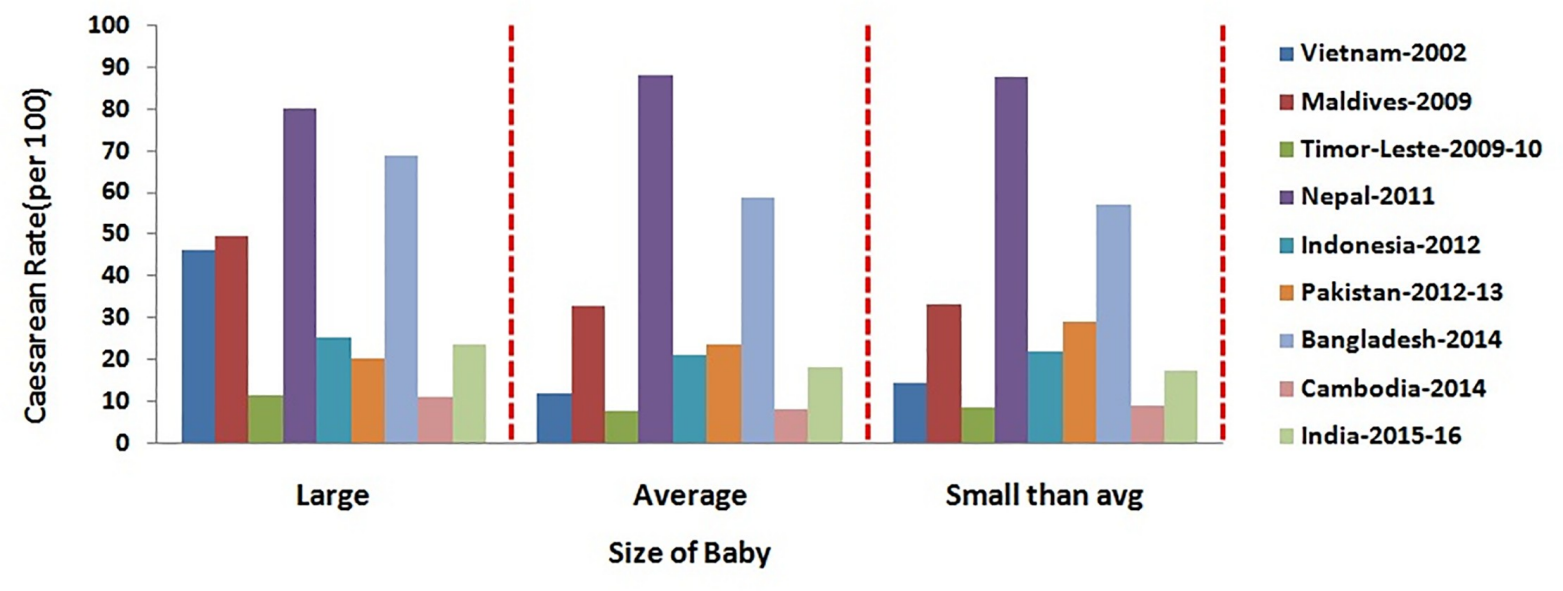
Caesarean rate based on size of the delivered child in South and South-East Asian countries.

**Fig 4:**
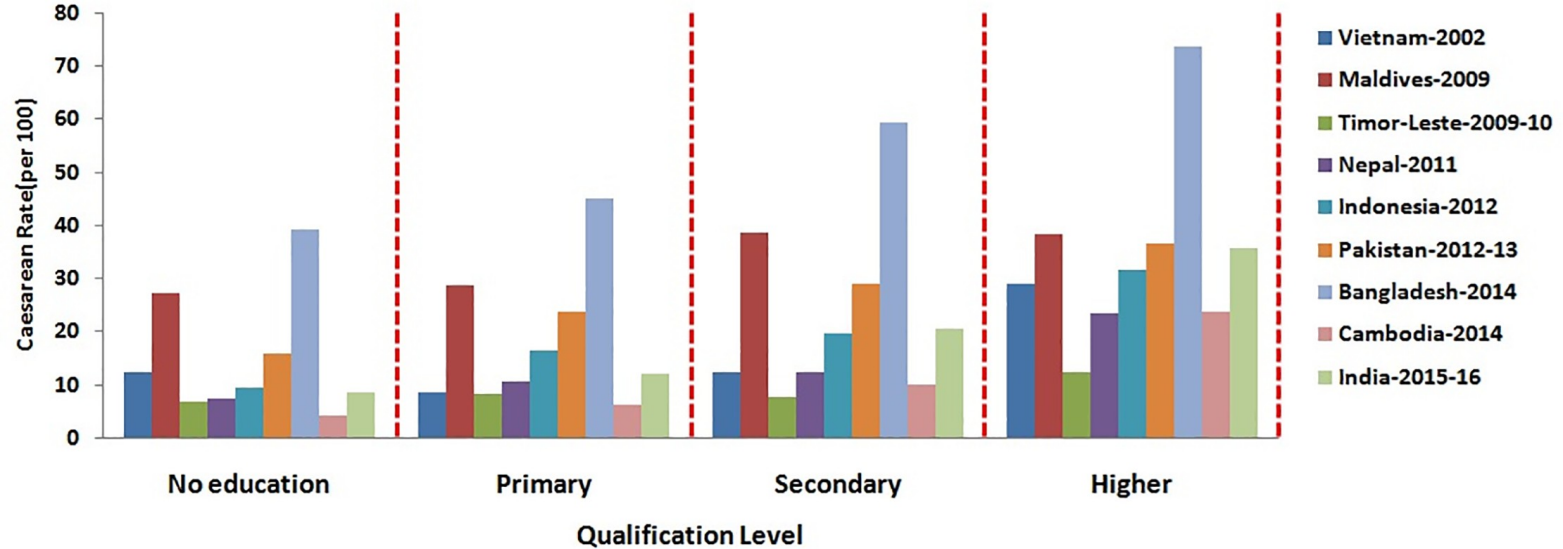
Caesarean rate based on mothers’ qualification in South and South-East Asian countries.

**Fig 5:**
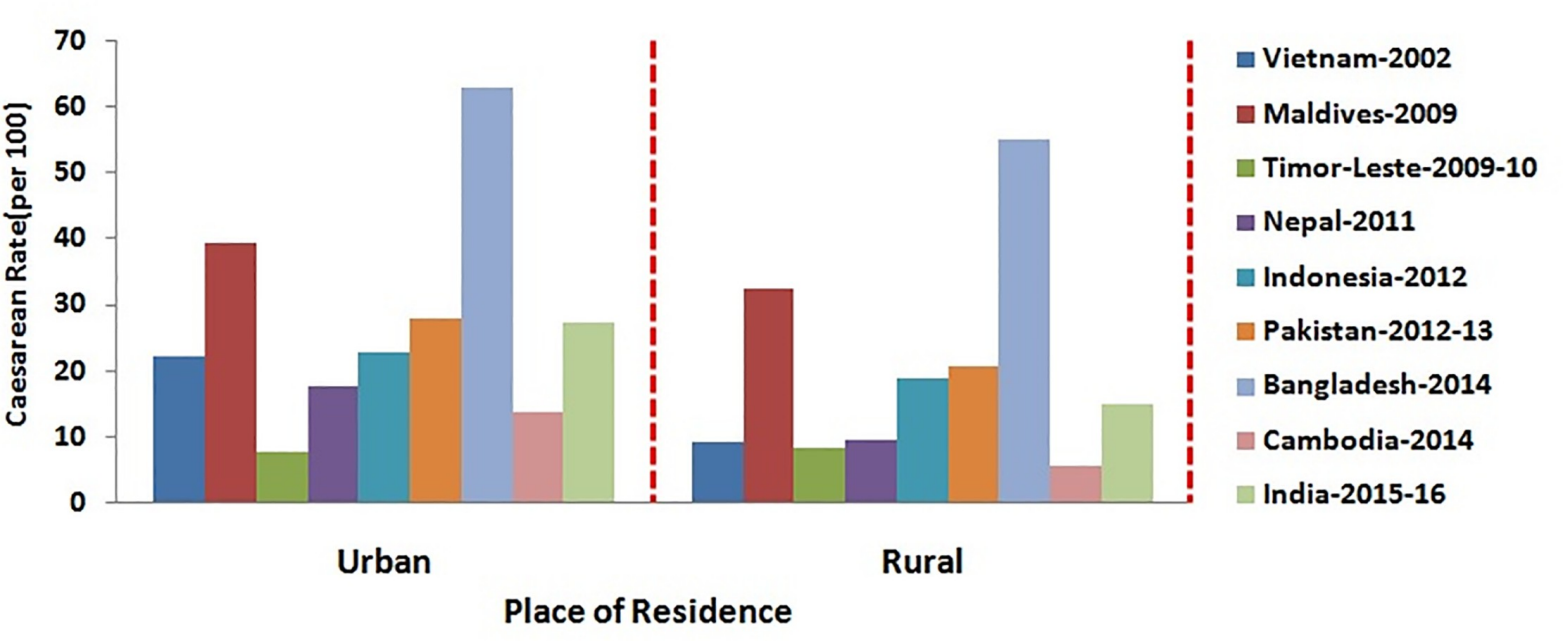
Caesarean rate based on mothers’ place of residence in South and South-East Asian countries.

**Fig 6:**
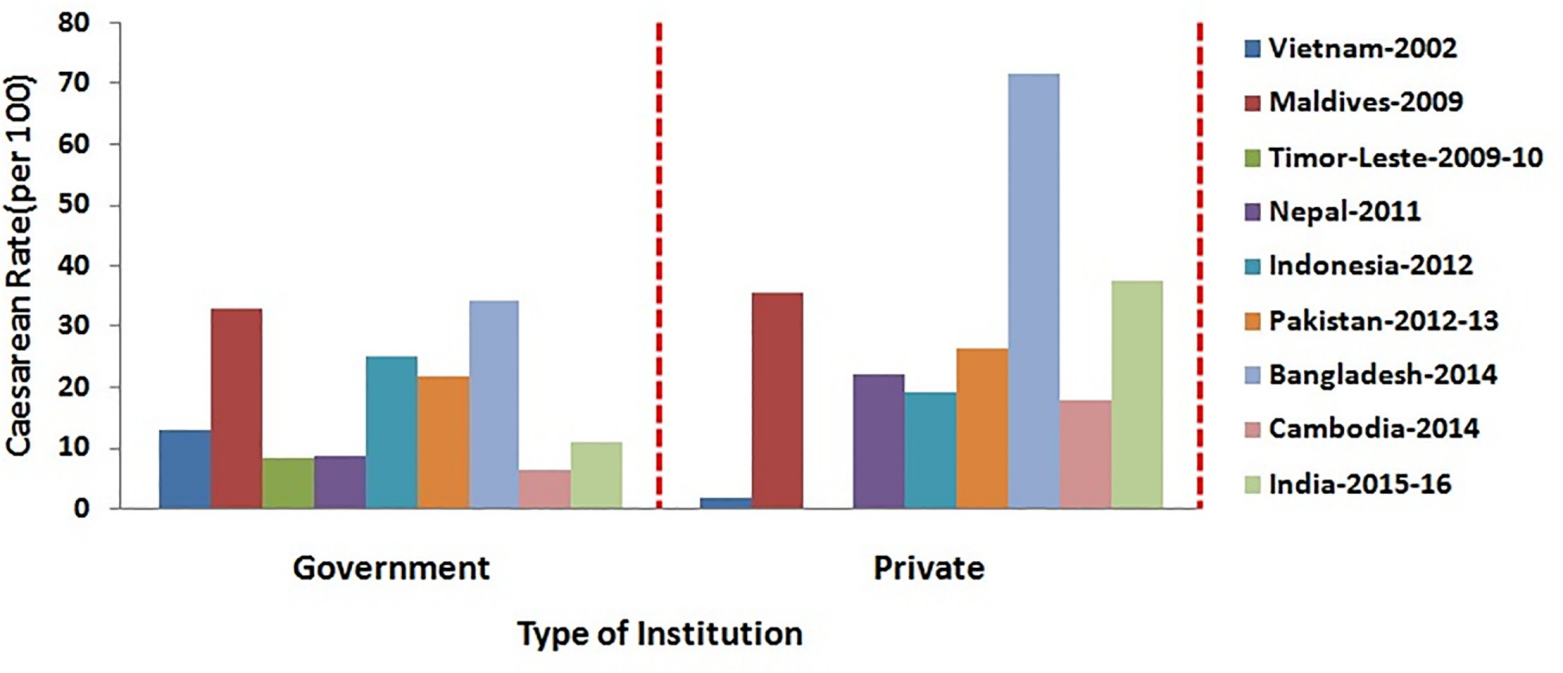
Caesarean rate based on choice of institution in South and South-East.

The percentage distribution of caesarean section among women based on their socio- demographic characteristics has been depicted in Figs 1–6. Fig 1 is showing a positive association of caesarean rate with the 15–39 age-grouped women, but on the contrary the caesarean rate among women belongs to 40–44 is comparatively low as compared to those belongs to 35–39. It also shows that the caesarean rates are more than 15% (WHO recommended) among the women aged 30 and above in all countries except Timor-Leste and Cambodia. Fig 2 depicts a negative association between the prevalence of caesarean delivery and the birth order of the child, i.e., chance to get caesarean section is high to women having birth order less than four. In countries Maldives and Bangladesh, caesarean rates are very high and found for all birth orders. From Fig 3, it has been found that caesarean is more preferred to women whose baby sizes are either very large or smaller than average. Irrespective of the size of the baby, caesarean rates in Maldives, Nepal, and Bangladesh have been found to be very high. Fig 4 depicts a positive association between caesarean section and women's education level. It is found in all countries that women who have a higher level of education are more predisposed towards caesarean childbirth. Caesarean sections among urban women, as depicted in Fig 5, were very high as compared to those of rural women except in Bangladesh, where rates are very high in women from both regions. Fig 6, depicts the caesarean sections among the women whose delivery occurred at private medical institutions which are very high compared to those delivered at government medical institutions.

### Determinants of prevalence of caesarean section

Tables 4 and 5 show the results of the bivariate and multivariate logistic regression analysis, respectively. The unadjusted analysis of multivariate logistic regression has revealed that maternal age, mother's education (except Vietnam and Maldives), choice of medical institutions (government and private) for childbirth (except Vietnam and the Maldives), birth order and place of residence (except the Maldives, Timor-Leste, and Bangladesh) have a significant effect on caesarean section. The variable baby size" has a significant effect on the prevalence of caesarean delivery (except Timor-Leste, Nepal, Indonesia, Bangladesh and Cambodia). The bivariate analyses applied in the study showed that maternal age, mother's education (except Timor-Leste), choice of medical facility for childbirth (except the Maldives), birth order and place of residence (except the Maldives) have a significant effect on the prevalence of caesarean section. The variable 'size of the baby' had been found to be insignificant in Timor-Leste, Nepal, Indonesia, Bangladesh, and Cambodia, while in the remaining countries viz.,Vietnam, Maldives, Pakistan, and India revealed a significant effect on the prevalence of caesarean delivery.

**Table 4:** Unadjusted odds ratio (OR) and 95% confidence interval (CI) for the risk of caesarean section corresponding to the associated factors in South and South-East Asian countries.

**Factor**	Vietnam	Maldives	Timor-Leste	Nepal	Indonesia	Pakistan	Bangladesh	Cambodia	India
**Age**	1.15* (1.10,1.20)	1.08* (1.06,1.10)	1.11* (1.07,1.15)	1.11* (1.07,1.15)	1.07* (1.06,1.08)	1.04* (1.03,1.06)	1.08* (1.05,1.11)	1.10* (1.08,1.12)	1.08* (1.08,1.09)
**Maternal Educn**									
No Education^R^	1	1	1	1	1	1	1	1	1
Primary	0.67 (0.22,2.08)	1.08 (0.85,1.37)	1.26 (0.67,2.37)	1.47 (0.92,2.34)	1.88 (0.97,3.64)	1.65* (1.37,1.99)	1.27 (0.83,1.96)	1.53* (1.01,2.31)	1.46* (1.39,1.53)
Secondary	1.01 (0.35,2.94)	1.68* (1.33,2.12)	1.13 (0.64,2.00)	1.76* (1.19,2.60)	2.37* (1.23,4.56)	2.18* (1.86,2.56)	2.25* (1.52,3.34)	2.48* (1.64,3.73)	2.75* (2.66,2.85)
Higher	2.97 (0.91,9.66)	1.68* (1.10,2.56)	1.95 (0.89,4.26)	3.77* (2.42,5.86)	4.48* (2.32,8.66)	3.09* (2.62,3.65)	4.35* (2.82,6.72)	7.12* (4.41,11.48)	5.90* (5.65,6.15)
**Institution**									
Government^R^	1	1	-	1	1	1	1	1	1
Private	0.11* (0.02,0.83)	1.12 (0.88,1.43)	-	3.02* (2.30,3.97)	0.71* (0.65,0.79)	1.28* (1.13,1.45)	4.82* (3.92,5.92)	3.29* (2.70,4.02)	4.94* (4.82,5.06)
**Birth order**									
1	7.32* (1.76,30.50)	2.42* (1.98,2.96)	2.15* (1.39,3.33)	2.35* (1.36,4.06)	1.43* (1.22,1.68)	2.48* (2.08,2.96)	2.49* (1.71,3.61)	1.73* (1.27,2.36)	7.03* (6.47,7.63)
2	5.41* (1.29,22.71)	1.92* (1.56,2.37)	1.13 (0.71,1.80)	2.17* (1.25,3.78)	1.27* (1.08,1.50)	1.85* (1.58,2.17)	2.06* (1.40,3.03)	1.22 (0.89,1.67)	5.10* (4.70,5.53)
3	3.97 (0.87,18.19)	1.32* (1.03,1.69)	1.15 (0.71,1.87)	1.87* (1.00,3.52)	1.21* (1.01,1.45)	1.99* (1.68,2.36)	1.65* (1.07,2.54)	1.04 (0.72,1.51)	2.69* (2.47,2.93)
4+^R^	1	1	1	1	1	1	1	1	1
**Residence**									
Urban	2.86* (1.95,4.18)	1.36* (1.12,1.65)	0.89 (0.64,1.25)	2.09* (1.59,2.73)	1.25* (1.13,1.39)	1.50* (1.33,1.69)	1.40* (1.16,1.68)	2.69* (2.23,3.25)	2.16* (2.11,2.21)
Rural^R^	1	1	1	1	1	1	1	1	1
**Baby’s size**									
Smaller than average	1.25 (0.69,2.28)	1.03 (0.84,1.26)	1.09 (0.68,1.75)	1.04 (0.72,1.50)	1.06 (0.92,1.22)	1.31* (1.13,1.52)	0.93 (0.73,1.19)	1.09 (0.80,1.49)	0.95* (0.91,0.98)
Avg. or more than avg^R^	1	1	1	1	1	1	1	1	1
Large	6.53* (2.15,19.78)	2.04* (1.40,2.97)	1.54 (0.75,3.16)	1.84 (0.83,4.04)	1.27* (1.01,1.60)	0.81 (0.33,2.00)	1.54 (0.75,3.16)	1.42 (0.94,2.15)	1.40* (1.34,1.45)

R stands for reference group.

*Statistically significant at 95% CI

**Table 5:** Adjusted odds ratio (OR) and 95% confidence interval (CI) for the risk of caesarean section corresponding to the associated factors in South and South-East Asian countries.

**Factor**	Vietnam	Maldives	Timor-Leste	Nepal	Indonesia	Pakistan	Bangladesh	Cambodia	India
**Age**	1.15*(1.10,1.20)	1.08*(1.06,1.10)	1.11* (1.07,1.15)	1.11* (1.07,1.15)	1.07* (1.06,1.08)	1.04* (1.03,1.06)	1.08* (1.05,1.11)	1.10*(1.081.12)	1.07* (1.06,1.08)
**Maternal Edu**									
No Education^R^	1	1	1	1	1	1	1	1	1
Primary	0.68(0.19,2.45)	1.12 (0.85,1.48)	1.54(0.81,2.94)	1.43(0.87,2.35)	1.94(0.99,3.78)	1.59* (1.31,1.92)	1.26 (0.78,2.03)	1.38(0.91,2.10)	1.20*(1.05,1.36)
Secondary	0.85(0.25,2.85)	1.32 (0.97,1.81)	1.40(0.77,2.53)	1.51(0.98,2.32)	2.50* (1.29,4.84)	1.91* (1.61,2.26)	1.94* (1.24,3.02)	1.82* (1.18,2.79)	1.28* (1.16,1.41)
Higher	1.20(0.31,4.63)	1.02 (0.63,1.65)	1.75(0.76,4.03)	2.06*(1.24,3.42)	3.95* (2.03,7.69)	2.32* (1.93,2.79)	2.6*(1.60,4.23)	3.19*(1.915.35)	1.56* (1.38,1.76)
**Institution**									
Government^R^	1	1	-	1	1	1	1	1	1
Private	0.14(0.02,1.06)	0.91 (0.71,1.18)	-	2.69*(2.03,3.58)	0.68* (0.61,0.75)	1.22* (1.07,1.38)	4.39* (3.55,5.43)	2.48* (2.00,3.07)	3.64* (3.55,3.74)
**Birth order**									
1	25.56* (5.44,60.12)	4.86* (3.58,6.59)	6.68* (3.70,12.08)	5.03* (2.50,10.14)	2.80* (2.28,3.43)	2.87* (2.30,3.57)	4.41* (2.60,7.47)	3.76* (2.515.61)	8.59* (7.82,9.42)
2	11.05* (2.44,50.08)	3.17* (2.41,4.18)	2.93* (1.65,5.21)	3.60*(1.89,6.87)	1.89* (1.57,2.27)	1.99* (1.65,2.41)	2.58* (1.61,4.12)	1.94* (1.34,2.81)	5.68* (5.19,6.21)
3	7.59* (1.57,36.74)	1.83* (1.39,2.42)	2.06* (1.21,3.50)	3.12*(1.57,6.20)	1.44* (1.19,1.74)	2.02* (1.68,2.44)	1.99* (1.23,3.24)	1.34* (0.90,1.98)	3.08* (2.75,3.31)
4+^R^	1	1	1	1	1	1	1	1	1
**Residence**									
Urban	1	1	1	1	1	1	1	1	1
Rural^R^	1.78* (1.15,2.77)	1.15 (0.93,1.43)	0.85(0.59,1.21)	1.85*(1.39,2.46)	1.13* (1.01,1.25)	1.15* (1.01,1.32)	1.14(0.93,1.40)	1.79* (1.45,2.21)	1.18* (1.10,1.26)
**Baby’s size**									
Smaller than average	1.07(0.55,2.06)	1.07 (0.87,1.31)	0.97(0.60,1.59)	1.15(0.78,1.69)	1.12(0.97,1.29)	1.48* (1.27,1.73)	1.02(0.78,1.33)	1.17(0.85,1.61)	1.06(0.98,1.15)
Avg. or moreavg^R^	1	1	1	1	1	1	1	1	1
Large	7.65* (2.19,26.74)	2.25* (1.53,3.31)	1.58(0.75,3.29)	1.67(0.72,3.89)	1.25 (0.98,1.58)	0.89 (0.35,2.23)	1.95 (0.85,4.43)	1.41 (0.912.17)	1.47* (1.27,1.70)

R stands for reference group.

*Statistically significant at 95% CI.

Women with higher education were more likely to have a caesarean section compared to those who have less education. Women who have opted for private institutions for delivery, compared to governmental medical institutions, were more likely to undergo a caesarean section. The order of birth showed a constant decrease in caesarean section. The place of residence showed that urban women, compared to their rural counterparts, have seen to be more likely to experience caesarean sections. The size of the infants has been found significant associated with caesarean section in countries viz., Vietnam, Maldives, Pakistan and India,which shows that women in these countries with a baby size outside of average reference size are more likely to give birth by caesarean section.

As caesarean is a surgical procedure and is possible in medical institutions, whereas women, whose delivery is not institutional, cannot be considered to be exposed for caesarean delivery and are not part of the population of interest. Corresponding to each selected country, the results revealed a shift towards institutional delivery over those of non-institutional deliveries, which indicate the effectiveness of health programs and mothers' increasing awareness of the importance of institutional delivery. It should also be noted regarding the Maldives that the prevalence of caesarean sections based on the total number of deliveries was 31.78%, which is quite close to the prevalence based on births in institutions, 33.14%. The reason for this closeness of estimates is that in the Maldives, 95.94% of births are institutional. In the case of Timor-Leste and Bangladesh, respectively, where 19.21% and 23.52% of births are in institutions. The prevalence based on the institutional births in Timor-Leste and Bangladesh has found to 4.45 and 5.2 times higher than those rates based on the total number of births, respectively. Our findings suggest that women with higher education are more likely to undergo caesarean as compared to uneducated women. The age of women was found to have a weak positive impact (the odds are slightly higher than one) on the risk of caesarean (i.e., every one-year increment on women's age, the risk of caesarean is approximately 1.1 times in women as compared to women with normal delivery). There are positive trends found for caesarean delivery in private hospitals. Our results indicate that odds for caesarean delivery in private hospitals as compared to government hospitals are very high. Results suggest that women with higher education are more likely to have caesarean sections than women who are uneducated. The age of women with low impact (odds are slightly higher than 1) on the risk of caesarean section. Results have indicated that odds for caesarean delivery in private hospitals as compared to government hospitals are very high.

**Fig 7:**
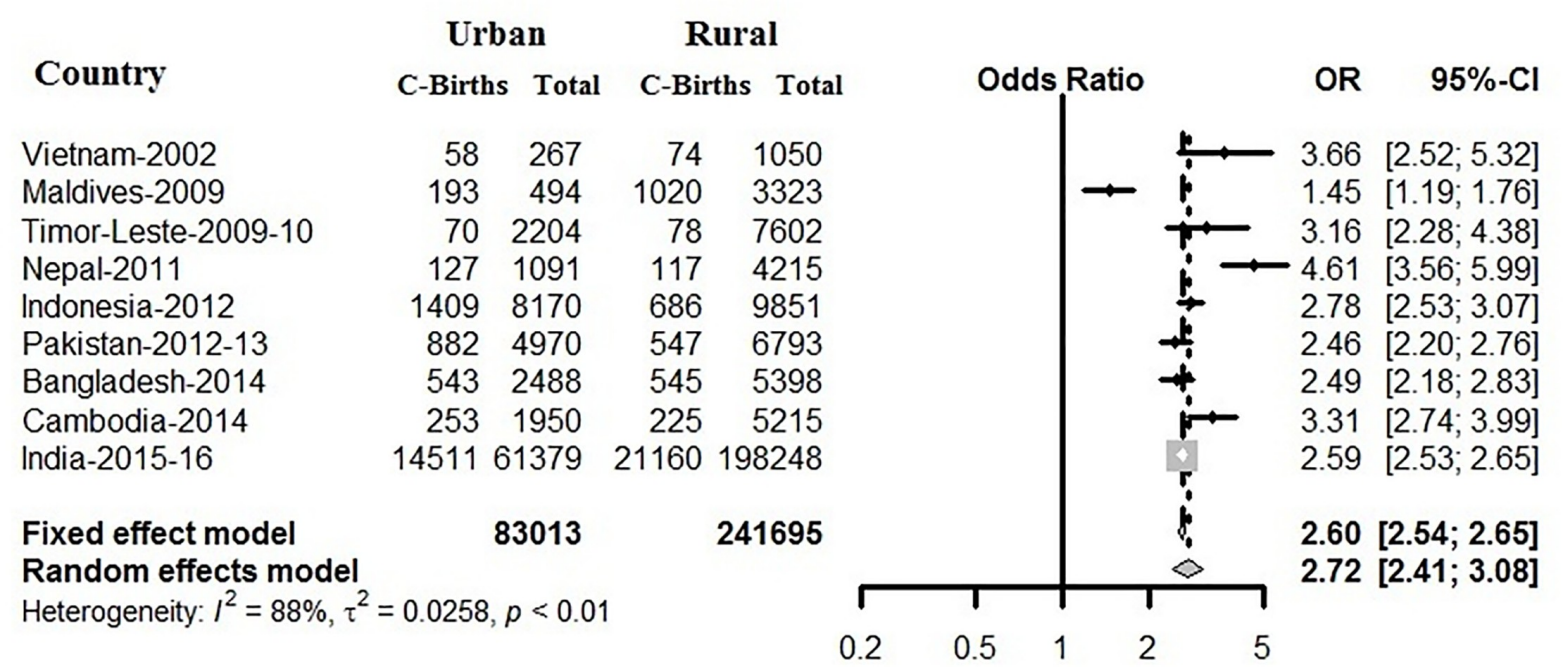
Sub-group meta- analysis on association of urban and rural caesarean rate based on both institutional and non-institutional births.

**Fig 8:**
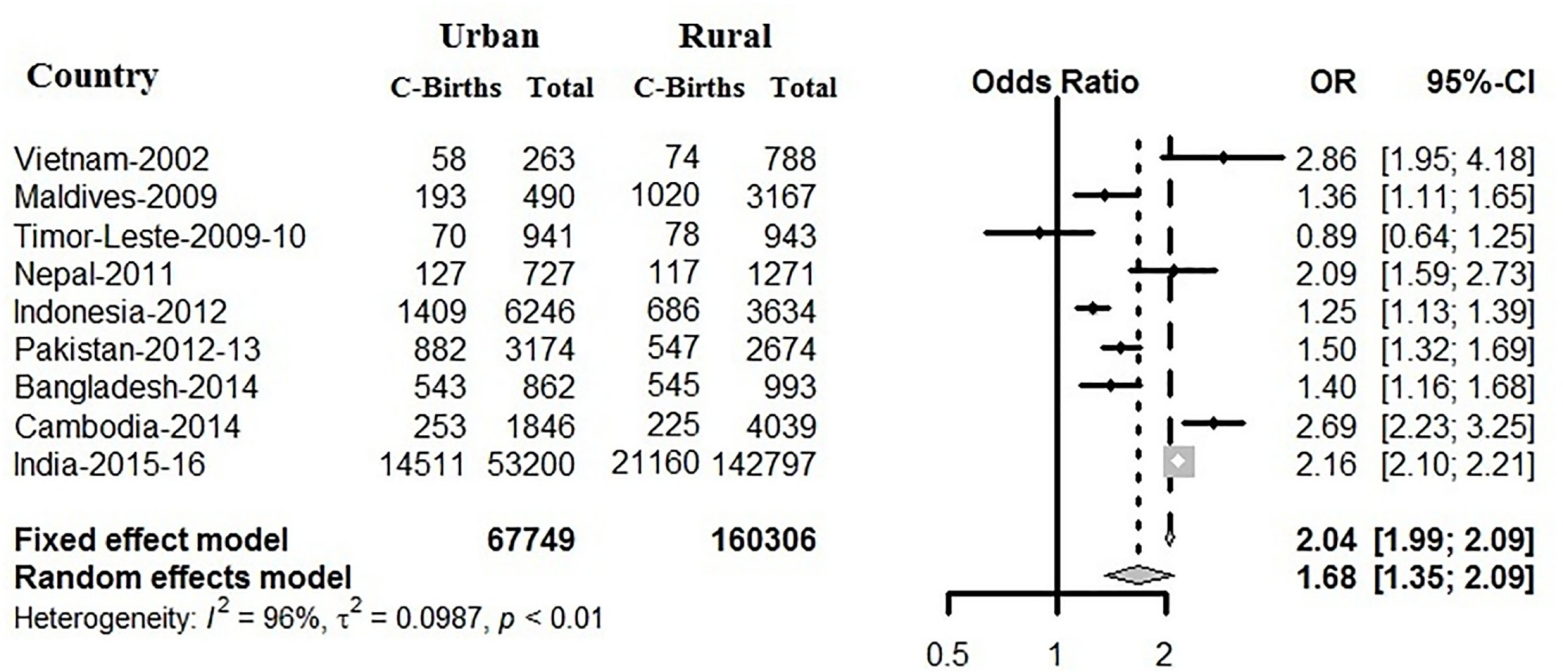
Sub-group meta- analysis on association of urban and rural caesarean rate based on institutional births.

Based on nine South and South-East Asian countries, where 3,24,708 births reported (both institutional and non-institutional) who had an overall C-section prevalence of 13% in Fig 7. All of the selected nine countries with an overall odds ratio (OR) of caesarean prevalence in urban as compared to rural was 2.60 (95% CI 2.54–2.65). Among 2,28,055 institutional births reported (Fig 8), with an overall prevalence of C-section 19%, except in Timor-Leste, the OR of caesarean prevalence in urban as compared to rural was more than one and are significant. Here, using Figs 7 and 8, we also meta-analysed caesarean data to demonstrate the pattern of C-section among urban and rural regions of different countries, by using the odds ratios and forest plot. Fig 7 examined the prevalence pattern of C-section in urban regions as compared to rural regions. The heterogeneity in the pattern of caesarean prevalence in urban and rural regions among countries can be confirmed by a high**I**^**2**^ value, which was found to be 88% based on combined institutional and non-institutional birth records, and 96% based on institutional births, and both of them have significant p-value. Due to this large and significant heterogeneity, pooled estimated of C-section in South and South-East Asian countries will not be appropriate, but confirms that in terms of prevalence of C-section all of the countries follow independent pattern.

There is a significant inclination in institutional deliveries in all selected countries, which indicates the effectiveness of women's awareness programs and programs. The main reason for this transition is that it reduces the risks and complications that occur during deliveries. This increase in the number of deliveries may be an important reason for caesarean delivery bias in countries viz., Maldives, Indonesia, Pakistan, Bangladesh and India,above the previous WHO recommended an optimal range of 10–15%. Having said this, the recommended rate might be higher if preventing serious morbidity is also taken into account. The prevalence of caesarean section is also examined for different socioeconomic covariate markers. The analysis shows that maternal age, maternal education, and birth order are significantly associated with caesarean delivery.

Of all the other determinants of the prevalence of caesarean delivery in any medical facility, the choice of place of birth viz., Government and private facilities may be a strong influence on the choice to undergo a caesarean section. Increases in the caesarean rates create a heavy burden on the health system[15] and also increases the risk of other health problems to both mother and baby, and unwanted caesarean delivery also puts a huge financial burden on the family economic status.

The limitations of the present study are that non-institutional births have not been taken into account to determine the extent of normal birth. The reason for non-institutional births could be attributed to fear of surgical proceeding involved in C-section or poor economical status or unavailability well equipped medical facility. Moreover, to under- stand the priority of C-section deliveries for further investigation of the reasons behind the prevalence, more relevant data on women and doctors' decision-making process for the safe child delivery and related risks, are needed. Due to unavailability of the reason for opting C-section among women having institutional births, further analysis about the situation where C-section is more preferable remained unexplored.

## 4. Conclusions

This study examines the prevalence of caesarean section in selected countries of South and South-East Asia, which is the top and third-largest populous region in the world. To represent the difference between rural and urban areas, the DHS datasets are considered so that the variation between the various facilities and the health and demographic indicators can be examined according to their place of residence. Both the logistic regression and the meta-analysis have shown a behavior change in rural and urban areas towards the adoption of a caesarean procedure. But in urban areas, rates are comparatively higher than in therural areas. The results showed that despite the disparity in the prevalence of caesarean section among rural and urban women, the percentages based on institutional births are completely different from those obtained using the information on total births. As a result, the government needs to develop better healthcare infrastructure and along with more antenatal care related schemes to reduce the risks associated with increased caesarean delivery.

## Supporting information

S1 File (PDF)
