# Supplementary material for: Prevalence and determinants of caesarean section in South and South-East Asian women
Source: PLoS One. 2020 Mar 12;15(3):e0229906. doi: 10.1371/journal.pone.0229906 (PMC7067459; doi:10.1371/journal.pone.0229906)
Supplement: S1 File — (PDF) [file pone.0229906.s001.pdf]

Gmail

Vivek Verma <vivverma456@gmail.com>

DHS Download Account Application

1 message

archive@measuredhs.com <archive@measuredhs.com> Wed, Jul 17, 2013 at 7:54 PM
To: vivverma456@gmail.com

See Attached Data Notes.

You have been authorized to download data from the Demographic and Health Surveys (DHS) on-line archive. This authorization is for unrestricted countries requested on your application.

All DHS data should be treated as confidential, and no effort should be made to identify any household or individual respondent interviewed in the survey.

The data sets must not be passed on to other researchers without the written consent of DHS. Users are requested to submit a copy of any reports/publications resulting from using the DHS data files. These reports should be sent to the attention of the DHS Data Archive: archive@measuredhs.com.

To begin downloading datasets, please login at http://www.measuredhs.com/data/dataset_admin/login_main.cfm. Once you are logged in, you may also edit your contact information, change your email/password, request additional countries or Edit/Modify an existing Description of Project.

If you are a first time user of DHS Data, please view the following videos on downloading and opening DHS data: http://www.measuredhs.com/data/Using-DataSets-for-Analysis.cfm#CP_JUMP_14039

Some more resources to help you analyze DHS data efficiently are available at: http://measuredhs.com/data/Using-Datasets-for-Analysis.cfm and on the MEASURE DHS User Forum at: http://userforum.measuredhs.com.

The files you will download are in zipped format and must be unzipped before analysis. Following are some guidelines:

After unzipping, print the file with the .DOC extension (found in the Individual/Male Recode Zips). This file contains useful information on country specific variables and differences in the Standard Recode definition.

Please download the DHS Recode Manual: http://measuredhs.com/publications/publication-dhsg4-dhs-questionnaires-and-manuals.cfm

The DHS Recode Manual contains the documentation and map for use with the data. The Documentation file contains a general description of the recode file, including the rationale for recoding; coding standards; description of variables etc. The Map file contains a listing of the standard dictionary with basic information relating to each variable.

It is essential that you consult the questionnaire for a country, when using the data files. Questionnaires are in the appendices of each survey's final report: http://measuredhs.com/publications/publications-by-type.cfm.

We also recommend that you make use of the Data Tools and Manuals: http://www.measuredhs.com/accesssurveys/technical_assistance.cfm.

DHS statistics can also be obtained using the STATcompiler tool: http://www.statcompiler.com. This tool allows users to select countries and indicators to create customized tables. It accesses nearly all of the indicators that are published in the final reports. Authorization is not needed to use the STATcompiler.

We highly recommend that dataset users register to participate in the MEASURE DHS User Forum at: http://userforum.measuredhs.com. The User Forum is an online community of DHS data users and contains discussions about many DHS analysis and dataset topics. Please search the contents of the forum, and if you do not see your question addressed, consider posting a new question for users to discuss.

If you have any questions or need assistance, please send an email to: archive@measuredhs.com.

MEASURE DHS Data Archive
ICF International
11785 Beltsville Drive
